# Fabrication of Concentrated Palm Olein-Based Diacylglycerol Oil–Soybean Oil Blend Oil-In-Water Emulsion: In-Depth Study of the Rheological Properties and Storage Stability

**DOI:** 10.3390/foods9070877

**Published:** 2020-07-03

**Authors:** Siou Pei Ng, Yih Phing Khor, Hong Kwong Lim, Oi Ming Lai, Yong Wang, Yonghua Wang, Ling Zhi Cheong, Imededdine Arbi Nehdi, Lamjed Mansour, Chin Ping Tan

**Affiliations:** 1 Department of Bioscience, Faculty of Applied Sciences, Tunku Abdul Rahman University College, Jalan Genting Kelang, Setapak, Kuala Lumpur 53300, Malaysia; ngsp@tarc.edu.my; 2 Department of Food Technology, Faculty of Food Science and Technology, Universiti Putra Malaysia, Serdang, Selangor 43400, Malaysia; sweet_appie@hotmail.com (Y.P.K.); hkwong_lim@yahoo.com.my (H.K.L.); 3 Department of Bioprocess Technology, Faculty of Biotechnology and Biomolecular Sciences, Universiti Putra Malaysia, Serdang, Selangor 43400, Malaysia; omlai@upm.edu.my; 4 JNU-UPM International Join Laboratory on Plant Oil Processing and Safety (POPS), Department of Food Science and Engineering, Jinan University, Guangzhou 510632, China; twyong@jnu.edu.cn; 5 Guangdong Research Center of Lipid Science and Applied Engineering Technology, School of Food Science and Engineering, South China University of Technology, Guangzhou 510640, China; yonghw@scut.edu.cn; 6 Department of Food Safety and Quality, School of Marine Science, Ningbo University, Ningbo 315211, China; lingzhicheong@yahoo.com; 7 Chemistry Department, College of Science, King Saud University, P.O. BOX 2455, Riyadh 11451, Saudi Arabia; imed12002@gmail.com; 8 Chemistry Department, El Manar Preparatory Institute for Engineering Studies, Tunis El Manar University, P.O. Box 244, Tunis 2092, Tunisia; 9 Zoology Department, College of Science, King Saud University, Saudi Arabia, P.O. Box 2455, Riyadh 11451, Saudi Arabia; lmansour@ksu.edu.sa

**Keywords:** diacylglycerol, soybean oil, emulsion, rheology, microstructure, emulsion stability

## Abstract

The present study focused on investigating the storage stability of oil-in-water (O/W) emulsions with high oil volume fractions prepared with palm olein-based diacylglycerol oil (POL-DAG)/soybean oil (SBO) blends at 25 °C. The incorporation of different ratios of oil blends significantly influenced (*p* < 0.05) the texture, color, droplet size distribution, and rheological parameters of the emulsions. Only emulsions incorporated with 10% to 20% POL-DAG in oil phase exhibited pseudoplastic behavior that fitted the Power Law model well. Furthermore, the O/W emulsions prepared with POL-DAG/SBO blends exhibited elastic properties, with *G’* higher than *G”*. During storage, the emulsion was found to be less solid-like with the increase in tan δ values. All emulsions produced with POL-DAG/SBO blends also showed thixotropic behavior. Optical microscopy revealed that the POL-DAG incorporation above 40% caused aggregated droplets to coalesce and flocculate and, thus, larger droplet sizes were observed. The current results demonstrated that the 20% POL-DAG substituted emulsion was more stable than the control emulsion. The valuable insights gained from this study would be able to generate a lot more possible applications using POL-DAG, which could further sustain the competitiveness of the palm oil industry.

## 1. Introduction

Generally, an oil-in-water (O/W) emulsion system consists of an oil phase, an aqueous phase, and an interface between the two immiscible phases where adsorption of the surface-active agents takes place [1]. Mayonnaise is a typical O/W emulsion example which may consist up to 80% of vegetable oil, followed by the remaining ingredients of water, vinegar, and egg yolk. With both hydrophilic and lipophilic properties within the same molecule, the emulsifiers found in the egg yolk stabilize the dispersed oil droplets via electrostatic interaction and by reducing the interfacial tension of the oil–water interface. Various oils were attempted for mayonnaise production, for instance, sunflower, canola, safflower, rice bran, olive, and soybean oil [2,3,4]. The application of soybean oil (SBO) in emulsions increased because of its economic advantages, nutritional properties, and availability. Linoleic acid, which appears to be the predominant unsaturated fatty acid in SBO (up to 50%), aids in reducing the total cholesterol level [5]. Furthermore, diacylglycerol (DAG) oils are widely known for their nutritional and health benefits in preventing obesity and other lifestyle-related diseases. In view of its health-promoting effects, it is also often used as a non-ionic emulsifier in food, pharmaceutical, and cosmetic industries. The DAG structure comprises a glycerol backbone with two fatty acids attached, in contrast to the three fatty acids of a triacylglycerol (TAG). DAGs can occur in two isomeric forms, namely, 1,2-DAG or 1,3-DAG, with the latter found to be more thermodynamically stable due to its steric effects [6]. DAG possesses higher polarity than TAG owing to the presence of the hydroxyl group. Thus, certain physical properties and the emulsification capability of DAG are found to be distinct from those of TAG [7]. DAG oil not only has higher emulsification ability but also has higher water-retention ability compared with those of TAG oil [8].

The production of purified palm olein-based diacylglycerol (POL-DAG) oil involves enzymatic glycerolysis and purification processes. POL-DAG was proposed as potential core ingredient in a wide array of food applications. Several studies reported the use of DAG, for instance, water-in-oil (W/O) emulsions like margarines and related products with improved overall properties of their emulsion systems were developed [9,10]. POL-DAG appears as a semi-solid state and easily solidifies at ambient temperature. To overcome this limitation, SBO is proposed to be blended with POL-DAG to modify the overall physicochemical properties for incorporation in O/W emulsion systems to produce sauces, mayonnaises, and salad dressings. The utilization of POL-DAG in blends with other oils/fats is believed to reduce the cost and also enhance the functionality of a variety of palm-based food products.

Emulsion instability leads to several emulsion destabilization mechanisms, for instance, gravitational separation, coalescence, creaming, flocculation, sedimentation, phase inversion, and Ostwald ripening. Generally, a stable mayonnaise is resistant against creaming due to the high oil volume fraction. This is because, with the high droplet density in the oil phase, the hydrodynamic hindrance imposed on oil droplet movement is increased [11]. Rheological data can provide useful insights in improving product formulations, especially in achieving desired flow characteristics. Extensive rheological studies were conducted on mayonnaise as it possesses complex viscoelastic rheological behaviors [12]. Nevertheless, the linear viscoelastic behavior was highlighted by several authors with reports on food development processes, who stated that this parameter is important in an emulsion system to gauge the emulsion stability [13,14]. Emulsions demonstrate non-Newtonian rheological behavior with apparent yield stress which can be fitted well by various models, for instance, Bingham, Herschel–Bulkley, power law, and Casson models [15]. Additionally, the thixotropic behavior of emulsions, which is a time-dependent rheology property, is also commonly described, in which the apparent viscosity decreases with increasing time (under a certain amount of shear stress) and the viscosity recovers when the emulsion is allowed to stand [16]. Therefore, these findings from reported studies suggested the importance of the aforementioned parameters in determining the emulsion stability and, thus, they must be thoroughly investigated, especially when new combinations or blends of oil are used as the dispersed phase, for instance, in developing a concentrated O/W emulsion, whereby the oil incorporation percentage is relatively high.

To date, reports of rheological studies on POL-DAG/SBO oil blends, as the oil phase in O/W emulsion systems, are scarce. The fabrication of the concentrated O/W emulsion in the present study is intended to simulate the mayonnaise-like emulsion model. Mayonnaise is a semi-solid emulsion which can consist of up to 80% vegetable oil as the dispersed phase. In this study, additives such as salt and sugar were not incorporated into the emulsion to avoid interference with the rheological behavior. Generally, the dispersed phase in emulsions normally consists of refined and/or fractionated oil, oil-soluble compounds, and surface active agents [17]. The oil phases in this study mainly consisted of triacylglycerol, monoacylglycerol (MAG), fatty acids, and the supplemented diacylglycerol. The main aim and novelty of the present work was to determine the suitability of POL-DAG/SBO oil blends in producing a stable concentrated O/W emulsion. This study will be able to provide a deeper understanding on the stability of O/W emulsions with the incorporation of POL-DAG oil as the dispersed phase. The emulsion stability could be elucidated through a series of analyses, such as texture and color properties, microstructure, particle size distribution, and rheological behaviors.

## 2. Materials and Methods 

### 2.1. Materials

SBO (SoyaLite brand), vinegar, and eggs were procured from a local supermarket (Selangor, Malaysia). For the production of POL-DAG oil, palm olein was provided by Sime Darby Sdn. Bhd. (Banting, Malaysia). Furthermore, Novozymes 435, the immobilized lipase from *Candida antarctica* was acquired from Novozymes A/S (Bagsvaerd, Denmark). For the preparation of the concentrated O/W emulsion, POL-DAG/SBO oil blends were prepared and incorporated as the dispersed phase, while deionized water was used as the continuous phase.

### 2.2. Production of POL-DAG and Oil Blending

By using Novozyme 435, POL-DAG oil was firstly produced via enzymatic glycerolysis [18]. The process was conducted in a pilot-scale packed-bed bioreactor system which consisted of a 10-L reaction vessel and a 6-L filtration vessel. After glycerolysis, the two-step purification process of DAG oil was conducted with the KD6 system (UIC, Alzenau-Hoerstein, Germany) using the short-path distillation method. In the first step, glycerol, MAG, and FFA were separated from the reaction mixture. In the second step, DAG was separated from the TAG and accumulated in the distillate vessel [18]. The resulting product was kept frozen at −20 °C for further use. 

For oil blending, POL-DAG and SBO were pre-heated at 70 °C, before being well homogenized and blended according to the desired ratios. Six different ratios of POL-DAG/SBO (0:100, 10:90, 20:80, 30:70, 40:60, and 50:50 (*w*/*w*)) were prepared to be incorporated as the dispersed phase in the concentrated O/W emulsion systems. The pure SBO (100% wt.) was used as the control sample. 

### 2.3. POL-DAG/SBO-Based O/W Emulsion Preparation

The ingredients used for the preparation of O/W emulsions were as follows: 70% oil blend, 20% water, 6% egg yolk, and 4% vinegar. For each batch, 200 g of emulsion sample was prepared. Firstly, the aqueous phase was prepared by mixing the egg yolk and vinegar with water. Next, the oil blend was slowly added to the continuous phase during the homogenization process. The emulsion was formed through homogenization at 12,500 rpm for 6 min at room temperature (25 ± 1 °C) using a high-speed Heidolph Diax 900 homogenizer (Schwabach, Germany). Each emulsion sample was prepared in three replications. All emulsions were transferred into individual glass bottles, tightly capped, and stored at room temperature (25 ± 1 °C) for 30 days. Sampling and analyses were conducted on day 1 and day 30 of storage. 

### 2.4. Textural Properties Analysis

The TA-XT2i texture analyzer (Stable Micro Systems Ltd., Surrey, UK) was used to characterize the texture profiles of the emulsions. A penetration test was performed using the spherical P/1SP probe (25.4 mm or 1 inch in diameter) and a 5-kg load cell. The emulsion samples were filled up to a depth of 75 mm in the stainless-steel container with 31.4 mm internal diameter and 85 mm height. The probe was set to penetrate 25 mm into the sample with 1 mm/s crosshead speed. Texture parameters such as the firmness and hardness value were obtained from the curves generated after the compression cycle. The analysis was performed in triplicate.

### 2.5. Color Analysis

A Konica Minolta CR-300 chromameter (Tokyo, Japan) was used to characterize the color of the emulsion samples using the CIE L*, a*, b* color system. A white calibration plate (L* = 97.10, a* = −0.07, b* = +1.97) was used as the standard for calibration. Referring to the color system, L* indicated the lightness, +a*/−a* represented the red/green coordinates, and +b*/−b* represented the yellow/blue coordinates. The color analysis of each emulsion samples was conducted in triplicate.

### 2.6. Particle Size Distribution

The particle size distribution of the emulsion samples was analyzed using a Mastersizer 2000 particle size analyzer (Malvern Instruments Ltd., Worestershire, UK) adopting the laser diffraction method. The obscuration rate was set at 5%, while the stirrer speed was maintained at 2500 rpm. The refractive indexes of SBO and water were fixed at 1.465 and 1.333, respectively. The background and sample measurement time were set at 10 s. To avoid multiple light scattering effects, dilution using distilled water to achieve a 0.05% droplet concentration was performed on all the emulsion samples. The particle size distribution was reported as volume-weighted mean diameter (*d*_4,3_), which was determined using the following formula: *d*_4,3_ = Σ*n_i_d_i_*^4^/Σ*n_i_d_i_*^3^, where *n_i_* represents the number of particles of diameter *d_i_*. The analysis was conducted in triplicate on all the emulsion samples.

### 2.7. Rheological Measurements

#### 2.7.1. Flow Behavior

All rheological measurements, namely, steady-shear measurements and oscillatory shear tests on emulsion samples, were performed using a Rheostress 6000 rheometer (Haake, Karlsruhe, Germany) coupled with Rheowin Job Manager software (Version 4.00). A sand-blasted cone sensor (C35/2° Ti; cone diameter = 35 mm, angle = 2°, and gap = 0.105 mm) and a measuring plate cover (MPC 35) were used for the analysis. The steady-shear rheological parameters were measured over a shear rate range of 0–100 s^−1^ within 2 min at 25 ± 0.1 °C. The collected rheological data were fitted using the Power Law model (Equation (1)), Herschel–Bulkley model (Equation (2)), and Casson model (Equation (3)) to examine the variations and to characterize the rheological behavior of the emulsions. The equations for each models are shown below.

(1) $$\sigma =K\gamma^{n},$$

(2) $$\sigma =\sigma_{0}+K{\dot{\gamma }}^{n},$$

(3) $$\sigma^{0.5}=\sigma_{c}{}^{0.5}+\eta_{c}{}^{0.5}{\dot{\gamma }}^{0.5},$$

where σ represents the shear stress (Pa), $\dot{\gamma }$ indicates the shear rate (s^−1^), *K* represents the consistency index (Pa.s^n^), *n* indicates the flow behavior index (dimensionless), $\sigma_{0}$ represents the yield stress (Pa), $\eta_{c}$ indicates the Casson plastic viscosity coefficient, and $\sigma_{c}$ represents the Casson yield stress. The apparent viscosity at 50 s^−1^ ($\eta_{a,50}$) was calculated from the magnitude of *K* and *n*. The rheological calculations were performed using Rheowin Data Manager software (Version 4.00).

#### 2.7.2. Thixotropic Profile 

A series of flow curves generated through the up/down time ramps were used to investigate the time-dependent thixotropic profile of the emulsion samples. A controlled shear rate at 25 °C was used to obtain the flow curves while shear stress was applied (shear rate range of 0–100 s^−1^ for 2 min, downtime of 2 min) to obtain the hysteresis loop. The thixotropic profile was quantified by area differences of the “hysteresis loop” between the ramp up and ramp down of a flow curve. The areas under the ramp up data points (A_up_) and under the ramp down data points (A_down_), as well as the hysteresis area (A_up_ − A_down_), were analyzed.

#### 2.7.3. Dynamic Viscoelastic Properties 

Oscillatory tests were frequently used to characterize the viscoelasticity of emulsions. Firstly, an amplitude sweep test was conducted on all emulsion samples. The linear viscoelastic (LVE) region was pre-determined in order to establish the range which the tests could be carried out without destroying the samples. A strain sweep from 0.1% to 70.0% with a frequency of 1 Hz were fixed to determine the stress range of LVE. The LVE range of all emulsions were found to be at strain values above 0.1568–0.5156%. Then, the frequency sweep was performed with a range of log 0.1 to 10 Hz at 0.005% strain. Several rheological parameters were monitored, for instance, the *G’* (storage modulus), *G”* (loss modulus), loss tangent (tan δ = *G”*/*G’*), and $\eta^{*}$ (complex viscosity) as a function of frequency. The analysis was performed in three replicates for each emulsion. The rheological properties were determined after the emulsions were stored for a day and 30 days. Based on visual observation, no phase separation was detected in the emulsion samples.

### 2.8. Microstructural Analysis 

A Nikon Eclipse 80i light microscope (New York, NY, USA) coupled with a Nikon charge-coupled device (CCD) camera (Kanagawa, Japan) and digital image processing software (NIS-Elements Basic Research, Nikon Instruments, New York, NY, USA) were used to observe the microstructure changes of the emulsion samples. Prior to observation under the microscope, the emulsion sample was gently dropped on a microscope slide and covered with a cover slip. The micrographs of the emulsion samples were observed and captured at a magnification of 60×.

### 2.9. Statistical Analysis

The collected experimental data were analyzed using Minitab version 16.0 (Sydney, Australia) statistical software. The results were tabulated as mean ± standard deviation. A one-way analysis of variance (ANOVA) with post hoc Tukey’s test was used to determine the significant differences between the mean values (*p* < 0.05).

## 3. Results and Discussion

### 3.1. Texture and Color Profiles

Table 1 shows the texture properties of all the emulsion samples after one day and 30 days of storage at 25 °C. The firmness and hardness of the emulsions containing the oil blends, from A to E, were found to increase with increased concentration of POL-DAG, which was mainly due to the variations in physicochemical properties of the samples and the control. No significant difference was observed among samples A and B with the control, although the former exhibited slightly higher firmness and hardness values compared to the control after storage for a day at 25 °C. However, samples C, D, and E were significantly (*p* < 0.05) firmer and harder than the control emulsion. Sample E, which consisted of 50% POL-DAG, possessed the highest firmness and hardness properties, whereas the control sample had the lowest firmness and hardness. After 30 days of storage, the firmness and hardness properties of samples B to E significantly increased (*p* < 0.05) to 42.38–160.13 N and 0.85–3.39 kg/s, respectively, compared to the control values. However, sample A and the control emulsion demonstrated contrary results, in which the firmness and hardness decreased. The findings are in accordance with a report which suggested that the decreases in the texture properties are most probably due to the droplet flocculation and/or coalescence during the initial storage period [14]. The texture of the emulsion samples containing 20% and more POL-DAG increased tremendously by 30 days of storage, which indicated that differences in their colloidal interactions imposed a dramatic effect on the texture of these food emulsions. Substituting SBO with 40% to 50% POL-DAG resulted in higher texture values of the emulsion samples. More energy was required to spread these O/W emulsions compared to that of the control; therefore, the concentration of 10% to 30% POL-DAG lowered the energy required to spread the emulsion, which is recommended in producing a variety of healthier O/W emulsion products. Overall, the textural behavior of all emulsion samples can be explained by their different dynamic viscoelasticity properties. 

The color changes of all emulsions after one day and 30 days of storage at 25 °C are tabulated in Table 1. The appearance of a mayonnaise product is mainly affected by the lightness, or the L*-value. [14]. Emulsion samples containing 20% to 50% of POL-DAG were slightly darker and more yellowish than the control sample, except for emulsion sample A, as shown by the slightly reduced L*-value, decreased a*-value (greenness), and increased b*-value (yellowness). Emulsion sample A was found to have the most similar color profile with the control sample. The lightness of the emulsion is often determined by the scattering effect, while the color is dependent on the absorption [19]. Sample E was significantly darker (*p* < 0.05) than the control sample, as demonstrated by the L*-value. The present findings are in good agreement with those reported by Chantraporchai, Clydesdale, and McClements [20], who suggested that the color changes of an emulsion from bright white to gray might indicated an increase in particle size distribution owing to the reduced light scattering effect. Thus, the lower L*-values were related to larger oil droplets that were present in samples D and E. After 30 days of storage, all emulsion samples showed an increment in L*-value and b*-value, but a decline in a*-value. All emulsion samples (A–E), including the control model, appeared to be more yellowish after storage for a month at 25 °C. This might be due to the significant (*p* < 0.05) increase in particle size with the increasing proportion of POL-DAG during storage, demonstrating that the effects of POL-DAG on color are strongly related to the oil droplet sizes. Generally, mayonnaise exhibits a shiny bright yellow color; however, in the present study, all model emulsions had a slightly yellow color that was mainly due to the pale yellowish color of purified POL-DAG oil present in the sample. 

### 3.2. Particle Size Distribution

**Figure 1:**
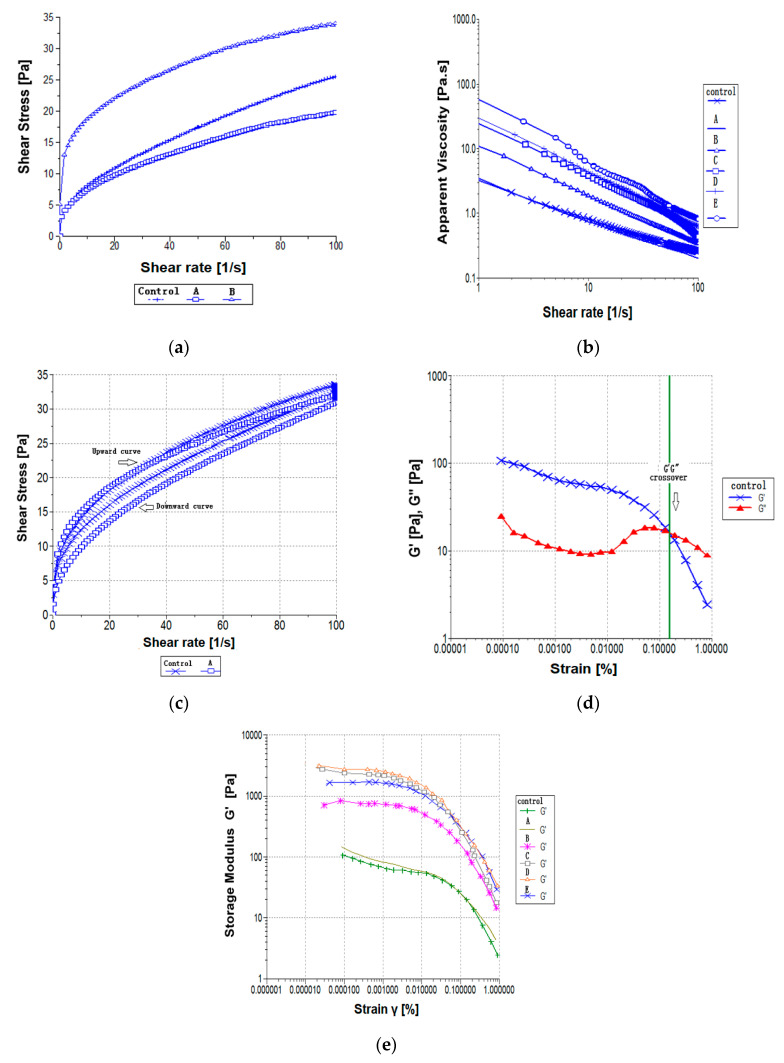
(**a**) Flow curves of all emulsion samples plotted with shear stress over shear rate; (**b**) dependence of viscosity on shear rate for all emulsion samples; (**c**) time-dependent behavior as a function of the increasing/decreasing shear rate of the flow curve against various emulsion samples; (**d**) storage modulus *G’* (Pa) and loss modulus *G”* (Pa) versus strain sweep; (**e**) storage modulus *G’* versus strain sweep. Abbreviations: Control: 100% soybean oil emulsion; A–E: emulsions containing 10–50% palm olein-based diacylglycerol (POL-DAG) substitution of soybean oil in the dispersed volume phase, respectively.

Table 2 shows the variations in the mean *d*[10], *d*[50], *d*[90] diameters, the span indices, and the volume-weighted mean diameters of the droplets in all emulsions after storage for one day and 30 days at 25 °C. The particle size of an emulsion is one of the key determinants in predicting the emulsion stability, as it provides some information about the dispersed phase at the interfaces. The representative particle size distributions of all emulsions containing oil blends were compared with the control. The particle sizes of all the emulsion samples exhibited bimodal distributions after storage for one day and 30 days (Figure 1). The mean particle diameters of the 30% to 50% POL-DAG emulsions after storage for one day were significantly (*p* < 0.05) larger compared to the other emulsion formulations and control, as shown by *d*[50] and *d*[90]. Furthermore, these emulsions also exhibited significantly (*p* < 0.05) larger particle sizes, with 90% of the overall particle size distribution consisting of mean particle diameters > 10 µm. The span index indicates the width distribution regardless of the median size [21]. The span index for the emulsion samples after one day of storage ranged between 1.70 and 1.98, whereas critical differences were observed after storage for 30 days, with the dispersion index increasing from 1.50 to 3.50. These vivid changes occurred particularly in samples B to E, as they showed significantly higher (*p* < 0.05) span indices than the control. The span index of sample B increased from 1.83 to 2.81 during 30 days of storage, indicating higher polydispersity. This phenomenon may be explained by the higher level of POL-DAG incorporation in the oil phase, which eventually increases the polydispersity of the emulsion, in accordance with the findings reported by Nor Hayati et al. [13]. As the oil substitution of SBO increased, the emulsion model contained increased amounts of saturated fatty acids with decreased amounts of unsaturated fatty acids. The significant changes in the particle size distribution in this study are mainly due to the proportion of the oil used in the formulations, particularly the different proportions of POL-DAG, rather than the effects of the aqueous phase. In terms of the volume-weighted mean diameters (*d*_4,3_), no significant difference (*p* > 0.05) was found for samples A and B compared to the control. However, samples C, D, and E exhibited significant (*p* < 0.05) larger particle sizes compared to the control.

During storage of emulsions, the aggregation of oil droplets due to the close proximity of droplets led to the formation of large flocs. Increasing the POL-DAG content from 20% to 30% decreased the mean diameter of the 50% size distribution *d*[50], whereas larger diameters were observed in samples A, D, and E. Samples B to E exhibited smaller-sized particles, particularly in *d*[10], and they had a bigger potential for an increase in the interface area compared to *d*[50] and *d*[90]. As for a mean diameter of 90% size distribution of *d*[90], samples D and E yielded the highest values, which were approximately >44.65 µm, indicating a larger interface in these emulsion models. Sample B exhibited the lowest mean diameter range of *d*[90], which was 16.78 µm and was insignificantly different (*p* > 0.05) compared to the control. However, the increase in the 90% size distribution *d*[90] of samples A to E significantly increased their particle size (*d*_4,3_), indicating that flocculation and coalescence were prevalent. During storage, the particle sizes of the emulsion samples increased gradually. Sample E exhibited the largest particle size among all the emulsion samples, with a sharp increment of 138.58% to 23.93 µm after 30 days of storage. The increased particle size was probably due to the coalescence phenomenon when the oil droplets came into close contact for an extended storage period [19]. The emulsion formulations with 20% to 30% POL-DAG incorporation showed no significant differences (*p* < 0.05) in their particle size in comparison with the control, and the sizes increased slightly during storage, with 25.20% and 3.55% increments, respectively. These two emulsions were found to be more stable because the droplet size only increased slightly during storage, and this stability might due to the presence of a gel network which subsequently delayed the oil droplets coalescence. The particle sizes (*d*_4,3_) of samples D and E were significantly (*p* < 0.05) larger than the control emulsion. Flocculation and coalescence occurred when the POL-DAG concentration increased from 40% to 50% in the emulsion model. Therefore, it was assumed that the oil droplets were prone to coalesce due to the larger droplet size which was determined in mean diameter range of 90%, while the span index also increased. In addition, higher yield stress values were calculated for samples D and E, suggesting oil droplet flocculation, which was further justified based on microstructure observation. The oil droplet diameter only acts as one of the indicators for the emulsion stability [20]. To understand emulsion droplet formation in an emulsion model, it is useful to perform a rheological study that will provide more in-depth information.

### 3.3. Rheological Tests

#### 3.3.1. Evaluation of the Flow Property and Thixotropic Profile

Steady-shear testing is commonly conducted to characterize the flow behavior of an emulsion. The shear stress (σ) versus shear rate ($\dot{\gamma }$) plots for all emulsion samples after one day of storage at 25 °C are shown in Figure 1a. Table 3 shows the consistency index (*K*), flow behavior index (*n*), yield stress ($\sigma_{0}$), Casson viscosity ($\eta_{c}$), and Casson yield stress ($\sigma_{c}$) results that were obtained from the flow curves that were fitted using Power Law, Herschel–Bulkley, and Casson models. Only the experimental data for emulsions A and B, as well as the control, were fitted using the Power Law model, Herschel–Bulkley model, and Casson model with high coefficients of determination (*R*^2^ ≥ 0.991) (Table 3). The Power Law model was applied to examine the rheological properties of the emulsion samples prepared with different POL-DAG/SBO oil blend ratios at 25 °C. However, the flow behavior of samples C, D, and E was not adequately fitted when using the Herschel–Bulkley model, as it generated negative yield stress values that were physically meaningless (Table 3). Moreover, the Casson model was utilized to describe the flow curves of all of the emulsion samples, but only samples A and B, as well as the control, were fitted using this model. The yield stress value is influenced by the magnitude of attractive forces between the droplets, in which weaker attractive forces result in lower yield stress [22]. The yield stress values that were obtained using the Casson model were significantly increased with the increased concentration of POL-DAG, ranging from 3.93 to 50.23 for the emulsion samples. Samples A and B had Casson yield stresses ($\sigma_{c}$) of 4.96 and 13.56, respectively. The results are in agreement with the study by Peressini et al. [23], who indicated the Casson yield stress ($\sigma_{c}$) values of four commercially available traditional or light mayonnaises as 40.6, 25.4, 23.4, and 19.4. The flow behavior index (*n*) indicates the fluid characteristics of a sample, where *n* = 1 represents Newtonian behavior, *n* < 1 represents shear-thinning behavior, and *n* >1 represents a shear-thickening behavior of a fluid. All of the emulsions studied showed non-Newtonian shear-thinning properties with low *n* values, ranging from 0.097 to 0.494. The flow behavior index and the consistency index of sample A were not significantly (*p* > 0.05) different, whereas emulsion samples B, C, D, and E were significantly lower (*p* < 0.05) compared to the control emulsions. Lower *n* values indicate increased shear-thinning behavior and more mutual entanglement. The shear-thinning behavior was most likely due to a dramatic shear-induced structural breakdown in food emulsions [19]. The reduction in apparent viscosity of the emulsions against time at a fixed applied shear was probably due to the deformation and/or reorganization of oil droplets/large flocs which were previously aggregated with weak forces. The apparent viscosity of the emulsion reached a plateau when the shear rate reached a certain level, which was probably due to the complete oil droplet disruption. The viscosity of emulsion samples provides important data for flow-process design, including stirring, pumping, and dispensing. Figure 1b shows the viscosity of all of the emulsion samples. The apparent viscosity at a shear rate of 50 s^−1^ in emulsion formulations with POL-DAG was found to increase linearly (*R*^2^ = 0.98), in the range of 0.32 to 1.46 (Pa·s) (Table 3). Higher viscosity values were found as the POL-DAG concentration increased from 30% to 50%, which reduced the flowing ability of the emulsions. The increasing viscosity of an emulsion can reduce the free motion of the oil droplets, thus retarding their creaming, flocculation, and coalescence [24]. The consistency index (*K*) indicates the viscous nature of an emulsion. A higher *K* value reflects a stronger emulsion network. Sample A had the lowest *K* value, indicating the least viscosity because of the high fluidity in the mixture. Samples C, D, and E were more highly viscous than the control. Sample E, which contained 50% POL-DAG, possessed the highest *K* value, which indicated that it had the strongest network among all samples. A higher oil content would contribute to a greater consistency index. This result confirms the previous finding which suggested that the oil volume fraction of the emulsion has great impact on the consistency index and the *K* value of an O/W emulsion [25].

Figure 1c shows the time-dependent thixotropic behavior with the ramp up/down curves exhibited by the control sample and sample A. The emulsion samples demonstrated thixotropic and shear-thinning behavior, with changes in their structure, throughout the studied shear rate range from 0 to 100 s^−1^. Thixotropy is a phenomenon when the viscosity of sample shows a reduction with the applied shear stress, but subsequently recovers when it is allowed to stand or when stress is removed (Mewis and Wagner 2009). The different thixotropic areas of all emulsions are shown in Table 4. The loop areas could be well explained by the energetic degradation of the structures that did not recover during the experimental period [26]. In all of the emulsion samples tested, the thixotropic area values were significantly (*p* < 0.05) different with a gradual increment in emulsion samples A–E, compared with that of the control. Emulsions that exhibit thixotropic behavior often contain oil droplets that are aggregated by weak forces [27]. Moreover, the degree of thixotropy of each emulsion sample was indicated by the hysteresis loop test, which increased as the oil concentration increased. In this study, the increase in the loop area of emulsion samples A to E could explain the gradual structure breakdown of the emulsion samples with the increasing shear time. The hysteresis loop between the curves generated the shear stress versus shear rate data and suggested that the sample flow was time-dependent [28]. However, after storage for 30 days, the thixotropic values of all samples except samples D and E tremendously decreased. Samples A and B exhibited thixotropic behavior similar to the control, whereas samples C, D, and E did not exhibit the same thixotropic profiles. These results are in agreement with the study reported by Štern et al. [29], stating that thixotropic characteristics were observed in mayonnaise samples with a shear rate range of 0 to 200 s^−1^.

#### 3.3.2. Strain Sweep Test

The oscillatory shear test is a non-destructive method used to analyze the viscoelastic behavior of emulsions. Figure 1d shows the variations in the storage and the loss modulus with the shear strain at a fixed frequency for all emulsions. The LVE region was determined by conducting small-amplitude strain sweep tests with a fixed frequency of 1 Hz over the strain range. Figure 1d illustrates the strain sweep measurements with the LVE region and the variations in the *G’G”* crossover values versus the strain of the control sample. Below the critical strain ($\gamma_{c}$) region, the structures appeared to be undamaged and the samples exhibited higher *G’* than *G”* values, indicating that they were highly structured. As the strain increases above the critical strain ($\gamma_{c}$) region, the network structure is disrupted and interactions between the colloidal particles change. The critical strain value is identified as the minimum strain above when the structure degradation of an emulsion starts to take place (for instance, the breakdown of large flocs into smaller units and/or the breakdown of a structuring agent) [30]. Once the critical strain region ($\gamma_{c}$) is exceeded, deformation of the emulsion structure and fragmentation of the larger flocs into smaller fragments begin [19]. The length of the LVE region is a measure of emulsion stability. Sample A and the control had a much shorter LVE region than did samples B, C, D, and E. Therefore, degradation of the emulsion structure occurred much more easily in sample A and the control sample. The increase in the viscoelasticity of samples B, C, D, and E of the emulsion model could strengthen the mutual attractive force between droplets, thus retarding flocculation and enhancing coalescence. The critical strain ($\gamma_{c}$) value of all samples fell in the range of 0.1568–0.5156%, but there was no consistent trend in the $\gamma_{c}$ values as the POL-DAG level increased. The $\gamma_{c}$ values of 0.1% to 0.5% indicate that the emulsion model was mainly an electrostatically stabilized system rather than a sterically stabilized system, in which the $\gamma_{c}$ values would range from 1% to 5%. The *G’* value, which exhibited a non-linear trend was found to decrease as the *G”* value increased. Each of the emulsions demonstrated a different *G’G”* crossover values even though the tests were performed using the same parameters. A drastic reduction in *G’* and *G”* values indicates complete structure breakdown of emulsions when greater stress is applied. Table 4 summarizes the *G’G”* crossover values of all emulsions after storage for a day and 30 days at 25 °C. The control sample and sample A exhibited *G’G”* crossover values at similar stress levels, at 16.36 and 15.69 Pa, respectively, whereas samples B to E exhibited significantly (*p* < 0.05) higher crossover values with a range of 61.09 to 174.62 Pa. Samples B exhibited stress sweep values similar to those reported by Fomuso et al. [31], who observed that the crossover values of structured lipid mayonnaise at 75 Pa indicated the stress levels of its gel structure. In this study, samples A and B exhibited lower crossover values and a high contribution of *G’* moduli to their viscoelastic properties. Due to their similar viscoelastic properties, samples A and B, as well as the control, displayed similar structural properties. Substitution of 10% to 20% POL-DAG for SBO caused the oil droplets to have much stronger mutual attractive forces that caused coalescence and flocculation. On the other hand, samples C, D, and E exhibited a greater degree of structural strength compared with other samples. A higher storage modulus value indicates that greater stresses are required to induce flow in an emulsion [32]. Samples C, D, and E exhibited much higher elastic moduli *G’* at low strain amplitudes compared to the control (Figure 1e), which resulted in a higher degree of internal structural formation in samples C, D, and E and their more solid-like behaviors. The substitution of SBO with 10 to 20% POL-DAG did not alter the gel structure of these emulsions. This result suggest that POL-DAG oil was vital in intensifying the elastic properties of the emulsions and enhanced the structured network strength in the emulsions. After 30 days of storage, the crossover values of samples A–D and the control were significantly (*p* < 0.05) decreased, but not sample E. Similar to the control, these results indicate that the gel structures present in the emulsion samples tolerate lower stress levels.

#### 3.3.3. Frequency Strain Sweep and Dynamic Viscoelastic Properties

**Figure 2:**
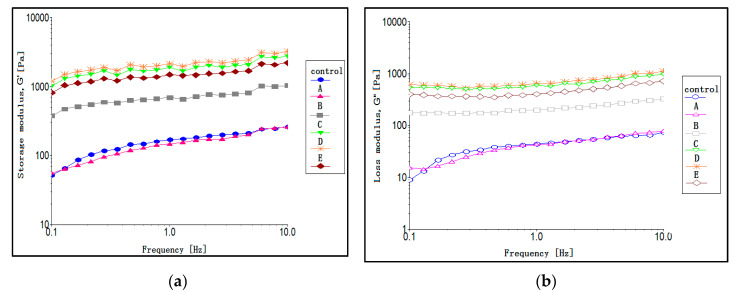

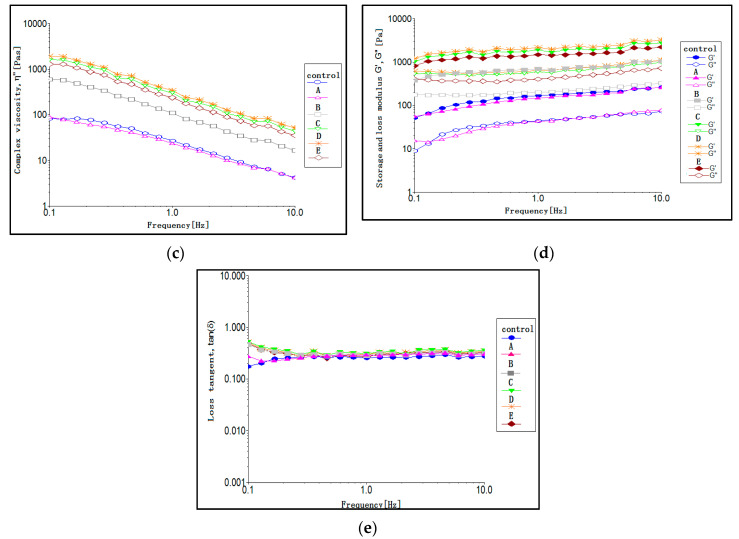
(**a**) Storage modulus *G’*, (**b**) loss modulus *G”*, (**c**) complex viscosity (η*), (**d**) *G’G”* versus frequency, and (**e**) loss tangent (tan δ) for all emulsion samples stored at 25 °C for one day. Abbreviations: Control: 100% soybean oil emulsion; A–E: emulsions containing 10–50% palm olein-based diacylglycerol (POL-DAG) substitution of soybean oil in the dispersed volume phase, respectively.

After the LVE regions of the samples were defined using the strain sweep assay, their structures were further characterized using a frequency sweep at a strain below the critical strain ($\gamma_{c}$) region. In this study, a low level of strain (0.005%) that was within the LVE region was applied in the dynamic frequency sweep tests. All emulsions stored overnight at 25 °C exhibited different trends of *G’* values. A higher *G’* value than *G”* characterized the network structures of all the emulsion samples (Figure 2a). The *G”* value increment occurred in parallel with the *G’* values, but relatively low *G’* moduli were observed in all emulsion samples (Figure 2b). The *G’* and *G”* values both increased with the increased frequency for all emulsions. Furthermore, all emulsions with 10% to 50% POL-DAG incorporation behaved as solid-like materials in which *G”* exceeded *G’*. This result is in agreement with the findings reported by Ma and Boye [33], who suggested that, when *G’* was greater than *G”*, a gel-like structure with a flocculated and entangled network existed, while *G”* higher than *G’* indicated the presence of non-flocculated or weakly flocculated emulsion systems. In addition, the emulsions in the present study exhibited a larger *G’* value, which might be due to the inter-particle droplet contacts in the emulsions. Interestingly, the increase in POL-DAG concentration from 10% to 50% increased the *G’* and *G”* values accordingly. On the other hand, the associated complex viscosity ($\eta^{*}$) values decreased accordingly in these emulsion samples, showing a frequency dependence (Figure 2c). According to Brummer [34], a stable emulsion which possesses strong internal strength normally exhibits a higher *G’* value than *G”* value, with both moduli appearing to be parallel throughout the frequency range studied, but with a slight increase in the slope when higher frequency is applied. Samples A–E and the control sample exhibited weak gel-like behavior due to the positive *G’* and *G”* slopes and higher *G’* values than *G”* values (Figure 2d). The *G’* values of samples C and D were higher than those of sample A and B. The magnitude of the *G’* and *G”* values increased in the sequence order of samples D > E > C > B >A > control. These results indicated that, for samples containing 10% to 50% POL-DAG, the variations in the blend ratios altered the *G’* value but did not change the relative elastic contribution to the viscoelasticity. The loss tangent (tan δ) is an important rheology parameter which measures the emulsion quality [35]. The emulsion strength can be reflected through its tan δ value; therefore, all the emulsion samples were analyzed at the same frequency of 1 Hz, and these data are tabulated in Table 4. The tan δ value directly indicated whether the samples exhibited viscous or elastic behavior, depending on whether the *G”*/*G’* ratio was smaller or larger than one, respectively. Here, the *G”*/*G’* ratio described the viscoelastic behavior of a sample; when the *G”*/*G’* value approached 1, the emulsion was becoming a liquid-like/viscous material, whereas a *G”*/*G’* value lesser than 1 indicated a solid-like/elastic behavior [35]. The tan δ values of all the emulsion samples fell in the range of 0.22 to 0.33 before storage, which indicated that the elastic behavior is more dominant in the samples than the viscous behavior (Figure 2e). However, after 30 days of storage, emulsions resulted in slightly higher loss tangent values, which were not greater than 0.37. This result suggests that the emulsion samples became less elastic during storage. Sample A had the highest tan δ value after storage, showing its high resistance to flow. A larger value of tan δ denotes that the emulsion samples flowed more easily. The tan δ values (0.29–0.33) of all emulsions prepared using POL-DAG/SBO were larger compared to the control emulsions, which indicates that they possessed stronger elastic gel-like properties than the control sample, which had a weak gel structure. A previous study reported that a mayonnaise sample showed weak gel properties when a frequency range of 0.1–10 Hz (0.63–62.83 rad·s^−1^) was applied [32]. The tan δ values were found to be closely related to the droplet sizes of the emulsions. The elastic gel-like behavior of the emulsion model can be attributed to the emulsifying capacity of POL-DAG.

### 3.4. Microstructural Analysis

**Figure 3:**
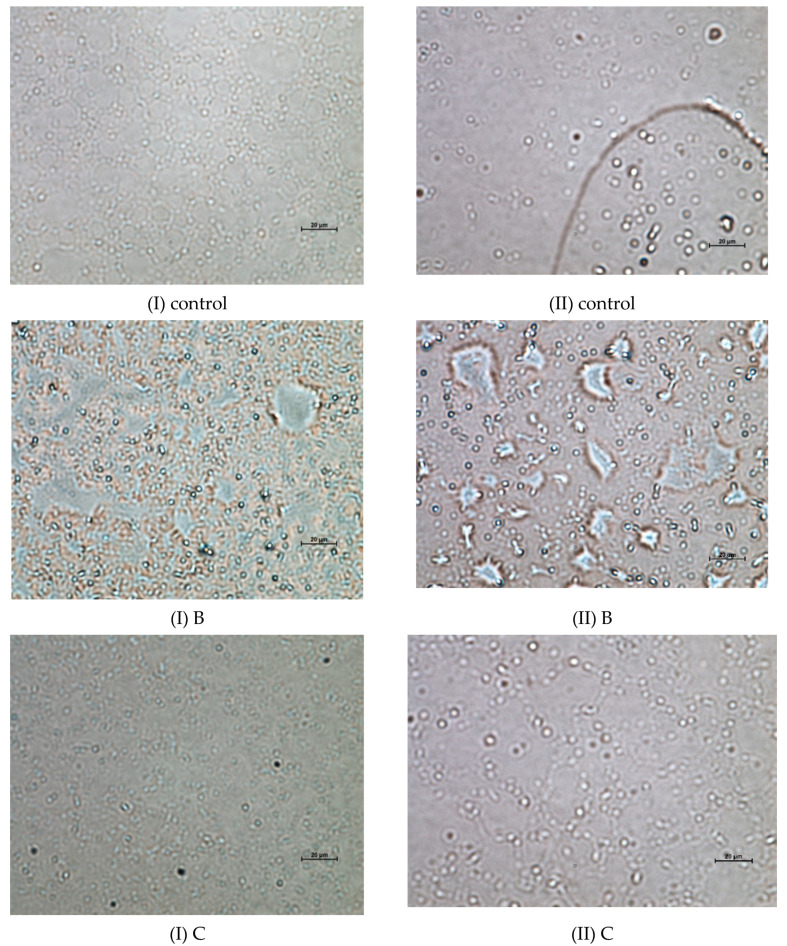

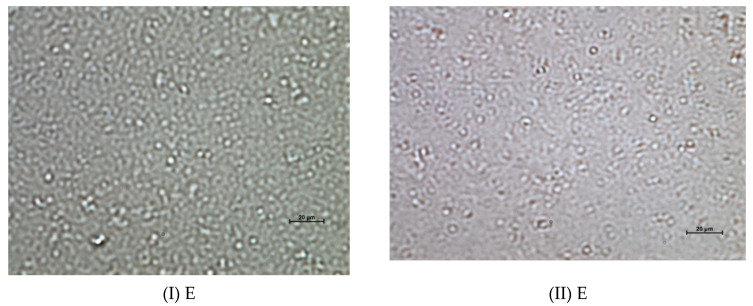
Microstructure of selected emulsions stored at 25 °C for (I) one day and (II) 30 days under a constant magnification of 60×. Control represents 100% soybean oil emulsion; B, C, and E represent emulsions containing 20%, 30%, and 50% palm olein-based diacylglycerol (POL-DAG) substitution of soybean oil in the dispersed volume phase, respectively.

Light microscopy was used to capture images of the microstructures of the emulsion samples that were stored overnight and for 30 days at 25 °C, as shown by some sample micrographs in Figure 3. The emulsion particle size distributions (as shown in Table 2) corresponded well with the observed microstructures. Many parameters of the microstructures of products determine their rheological properties, which include the interactions, junctions, and properties of the structural components, as well as the interfacial behaviors or structures of the phases [36]. The homogenization technique utilized often critically affects the microstructures of emulsions. In this study, emulsions which were subjected to high-shear homogenization were found to be polydispersed as they contained droplets with different sizes. To produce a monodispersed emulsion with uniform droplet size distribution would be almost impossible without the usage of a mill or high-pressure homogenizer, which could further break down the larger droplets into smaller sizes [14]. However, homogenization is not the only factor that affected the emulsions’ microstructures. Indeed, different concentrations of POL-DAG incorporation greatly affected the microstructures of the emulsions that were prepared in this study. The microstructures of samples A–E containing 10% to 50% POL-DAG substituted for SBO showed that their spherical oil droplets were so closely packed that they were hardly able to move compared with those of the 100% SBO control. The control emulsion contained oil droplets with a droplet size distribution range of 0.15 to 9.91 µm. Apparently, the oil droplets in the control sample and samples A, D, and E aggregated to form larger droplets after storage for 30 days. As a concentrated O/W emulsion model, all of the emulsions are flocculated because the surfaces of neighboring droplets are in close contact, in which a three-dimensional network of aggregated droplets is formed [14]. In a more concentrated system, the droplet aggregates may lose their identity, developing semi-continuous floc structures that permeate the entire system [37].

The size of the droplets in samples B and C decreased during storage for 30 days (*p* < 0.05), and their droplets became mutually entangled. In the emulsion model, the strong interactions among the droplets of samples B and C required a higher yield stress to allow emulsion flow. In samples D and E, the increase in oil droplet coalescence and flocculation with the increasing content of the POL-DAG oil blend caused an increase in the rate of structural breakdown.

## 4. Conclusions

This study clearly demonstrated that the different levels of 10% to 50% POL-DAG incorporation in the dispersed phase fraction altered the particle size distribution, color, microstructures, texture, and rheological properties of the concentrated O/W emulsion model, and that the effects highly depended on the level of POL-DAG substitution. It was shown in a model emulsion that replacing the SBO with POL-DAG at the 20% level provided the best emulsion stability during storage for 30 days at 25 °C, suggesting that it could be used for food product development. Increasing the substitution of POL-DAG above 20% contributed to increasing the particle size and increasing the viscoelasticity, thereby leading to emulsion destabilization. In addition, the hysteresis loop was larger in the samples containing 30% to 50% POL-DAG and smaller in those containing 10% to 20% POL-DAG, implying more and less structural damage, respectively. This study also showed that rheological parameters could be used as a guideline to investigate and select the optimal oil blend level for preparing a stable emulsion sample. Further study of the O/W emulsion model properties with 20% POL-DAG incorporation is recommended to develop it into a food product such as mayonnaise and to extend the results of this investigation to reach specific conclusions. Overall, an emulsion containing a POL-DAG/SBO oil blend with enhanced techno-functional properties and nutritional values will be able to spark interest among food industries owing to its wide potential with possibilities in innovative applications.

## Figures and Tables

**Table 1:** Textural and color profiles of concentrated oil-in-water (O/W) emulsion samples stored at 25 °C for one day and 30 days.

	Storage Period									
Samples	One-Day		30 Days		One-Day			30 Days		
	Texture Properties				Color Profiles					
POL-DAG/SBO	Firmness (N)	Hardness (kg/s)	Firmness (N)	Hardness (kg/s)	L*	a*	b*	L*	a*	b*
**Control (0:100)**	13.94 ± 0.09 ^c^	0.34 ± 0.01 ^c^	11.39 ± 0.06 ^e^	0.24 ± 0.01 ^c^	82.88 ± 0.05 ^b^	−1.99 ± 0.02 ^a^	9.47 ± 0.10 ^c^	86.77 ± 0.01 ^a^	−2.50 ± 0.01 ^a^	14.40 ± 0.01 ^f^
**A (10:90)**	15.59 ± 0.92 ^c^	0.37 ± 0.02 ^c^	13.81 ± 0.20 ^e^	0.35 ± 0.01 ^c^	83.07 ± 0.06 ^a^	−1.95 ± 0.02 ^a^	10.29 ± 0.10 ^b,c^	83.86 ± 0.01 ^e^	−2.75 ± 0.01 ^b^	16.44 ± 0.02 ^b^
**B (20:80)**	15.33 ± 0.16 ^c^	0.36 ± 0.01 ^c^	42.38 ± 2.30 ^d^	0.85 ± 0.70 ^b,c^	82.44 ± 0.01 ^c^	−2.27 ± 0.03 ^b^	11.27 ± 0.10 ^a,b^	83.06 ± 0.02 ^f^	−3.38 ± 0.03 ^e^	16.95 ± 0.27 ^a^
**C (30:70)**	44.51 ± 2.69 ^b^	0.95 ± 0.04 ^b^	75.97 ± 2.40 ^c^	1.68 ± 0.25 ^b^	82.41 ± 0.03 ^c^	−2.31 ± 0.02 ^b^	11.32 ± 0.10 ^a,b^	84.80 ± 0.01 ^d^	−3.08 ± 0.03 ^d^	16.31 ± 0.21 ^c^
**D (40:60)**	44.85 ± 0.47 ^b^	0.99 ± 0.02 ^b^	133.85 ± 6.80 ^b^	2.78 ± 0.44 ^a^	82.28 ± 0.02 ^d^	−2.23 ± 0.03 ^b^	10.47 ± 0.10 ^b,c^	85.00 ± 0.03 ^c^	−3.00 ± 0.02 ^c^	15.79 ± 0.10 ^d^
**E (50:50)**	55.23 ± 0.99 ^a^	1.06 ± 0.03 ^a^	160.13 ± 12.42 ^a^	3.39 ± 0.64 ^a^	81.70 ± 0.02 ^e^	−2.32 ± 0.06 ^b^	11.62 ± 0.10 ^a^	85.32 ± 0.02 ^b^	−2.77 ± 0.02 ^b^	15.94 ± 0.12 ^e^

POL-DAG: palm olein-based diacylglycerol oil; SBO: soybean oil; L*: lightness (+)/darkness (−); a*: redness (+)/greenness (−); b*: yellowness (+)/blueness (−). Data are expressed as the mean ± standard deviation. Mean values with different superscripts (a–f) in the same column are significantly different at *p* < 0.05 based on ANOVA and Tukey’s tests.

**Table 2:** Droplet mean diameter, dispersity index (span), and volume-weighted mean of concentrated oil-in-water (O/W) emulsion samples stored at 25 °C for one day and 30 days.

Storage Period										
Samples	One-Day					30 Days				
POL-DAG/SBO	*d*[10]	*d*[50]	*d*[90]	Span Index	*d*_4,3_ (µm)	*d*[10]	*d*[50]	*d*[90]	Span Index	*d*_4,3_ (µm)
Control (0:100)	0.15 ± 0.03 ^b^	5.74 ± 0.23 ^c^	9.91 ± 0.03 ^e^	1.70 ± 0.01 ^c^	5.78 ± 0.03 ^c^	2.29 ± 0.13 ^b^	7.65 ± 0.92 ^c^	13.73 ± 0.45 ^d^	1.50 ± 0.04 ^d^	8.43 ± 0.36 ^c^
A (10:90)	0.18 ± 0.02 ^a,b^	5.43 ± 0.01 ^c^	10.03 ± 0.65 ^e,d^	1.81 ± 0.03 ^b,c^	5.46 ± 0.35 ^c^	2.94 ± 0.17 ^a^	17.14 ± 1.73 ^a^	30.60 ± 6.06 ^b^	1.61 ± 0.15 ^d^	17.08 ± 2.97 ^b^
B (20:80)	0.25 ± 0.07 ^a^	5.67 ± 0.25 ^c^	10.61 ± 0.60 ^d^	1.83 ± 0.03 ^b,c^	6.19 ± 0.50 ^c^	0.09 ± 0.01 ^c^	5.95 ± 0.11 ^d^	16.78 ± 0.50 ^c,d^	2.81 ± 0.34 ^c^	7.75 ± 0.16 ^c^
C (30:70)	0.17 ± 0.02 ^b^	8.61 ± 0.65 ^b^	15.84 ± 0.69 ^c^	1.82 ± 0.05 ^b,c^	8.17 ± 0.94 ^b^	0.09 ± 0.01 ^c^	6.14 ± 0.62 ^c,d^	21.25 ± 0.50 ^c^	3.45 ± 0.54 ^b^	8.46 ± 0.33 ^c^
D (40:60)	0.17 ± 0.02 ^b^	8.81 ± 0.66 ^b^	17.09 ± 0.79 ^b^	1.92 ± 0.04 ^a,b^	9.09 ± 0.90 ^a,b^	0.12 ± 0.01 ^c^	13.23 ± 1.21 ^b^	46.45 ± 5.90 ^a^	3.50 ± 0.87 ^a^	22.58 ± 2.63 ^a^
E (50:50)	0.16 ± 0.02 ^b^	9.96 ± 0.99 ^a^	19.77 ± 0.84 ^a^	1.98 ± 0.12 ^a^	10.03 ± 1.24 ^a^	0.14 ± 0.02 ^c^	13.78 ± 1.19 ^b^	44.65 ± 5.95 ^a^	3.23 ± 0.55 ^b,c^	23.93 ± 2.67 ^a^

POL-DAG: palm olein-based diacylglycerol oil; SBO: soybean oil; *d*[10], *d*[50] and *d*[90]: size values corresponding to the cumulative distribution at 10%, 50%, and 90%, respectively; *d*_4,3_: volume-weighted mean. Data are expressed as the mean ± standard deviation. Mean values with different superscripts (a–e) in the same column are significantly different at *p* < 0.05 based on ANOVA and Tukey’s tests.

**Table 3:** Comparison of three rheological fitting model parameters on flow curves of concentrated oil-in-water (O/W) emulsion samples stored at 25 °C for one day.

Samples	Power Law Model			Herschel–Bulkley Model				Casson Model			$\eta_{a}\mathrm{at}\;50\;s^{-1}\;(\mathrm{Pa}\cdot s)$
POL-DAG/SBO	*K* (Pa·s)	*n*	*R* ^2^	*K* (Pa·s)	*n*	$\sigma_{0}\;(\mathrm{Pa})$	*R* ^2^	$\sigma_{c}$	$\eta_{c}$	*R* ^2^	$\eta_{a,50}$
**Control (0:100)**	2.72 ± 0.50 ^d^	0.494 ± 0.024 ^a^	0.999	1.98 ± 0.15 ^c^	0.567 ± 0.032 ^a^	1.59 ± 0.16 ^a^	0.999	3.93 ± 0.57 ^d^	0.111 ± 0.010 ^a^	0.996	0.38 ± 0.03 ^e^
**A (10:90)**	2.95 ± 0.55 ^d^	0.454 ± 0.024 ^a^	0.997	2.35 ± 0.17 ^c^	0.461 ± 0.022 ^b^	0.67 ± 0.04 ^a^	0.999	4.96 ± 0.60 ^d^	0.062 ± 0.040 ^b^	0.992	0.32 ± 0.03 ^e^
**B (20:80)**	11.29 ± 1.60 ^c^	0.263 ± 0.021 ^b^	0.998	12.66 ± 0.70 ^c^	0.239 ± 0.015 ^c^	2.01 ± 0.24 ^a^	0.998	13.56 ± 1.58 ^c^	0.065 ± 0.042 ^b^	0.991	0.63 ± 0.02 ^d^
**C (30:70)**	27.05 ± 3.70 ^b^	0.135 ± 0.015 ^c,d^	0.859	619.27 ± 66.60 ^b^	0.010 ± 0.005 ^d^	−593.30 ± 115.80 ^b^	0.981	30.77 ± 0.90 ^b^	0.025 ± 0.009 ^c^	0.854	1.04 ± 0.03 ^c^
**D (40:60)**	30.83 ± 2.90 ^b^	0.158 ± 0.018 ^c^	0.954	651.60 ± 72.40 ^b^	0.120 ± 0.010 ^d^	−617.40 ± 108.60 ^b^	0.915	33.96 ± 0.80 ^b^	0.054 ± 0.001 ^b^	0.838	1.23 ± 0.03 ^b^
**E (50:50)**	52.75 ± 6.14 ^a^	0.097 ± 0.002 ^d^	0.916	702.60 ± 122.49 ^a^	0.002 ± 0.001 ^d^	−715.23 ± 122.90 ^c^	0.834	50.23 ± 1.99 ^a^	0.050 ± 0.001 ^b^	0.819	1.46 ± 0.06 ^a^

POL-DAG: palm olein-based diacylglycerol oil; SBO: soybean oil; *K*: consistency index; *n*: flow behavior index; $\sigma_{0}$: yield stress;$\;\eta_{c}$: Casson viscosity; $\sigma_{c}$: Casson yield stress; $\eta_{a}$: apparent viscosity; *R*^2^: coefficient of determination. Data are expressed as the mean ± standard deviation. Mean values with different superscripts (a–e) in the same column are significantly different at *p* < 0.05 based on ANOVA and Tukey’s tests.

**Table 4:** Thixotropic area, *G’G”* crossover, storage modulus (*G’*), loss modulus (*G”*), and loss tangent (tan δ) of concentrated oil-in-water (O/W) emulsion samples stored at 25 °C for one day and 30 days.

Storage										
Samples	One-day					30 days				
POL-DAG/SBO	Thixotropy Area (Pa/s)	*G’G”* Crossover (Pa)	*G’* (Pa)	*G”* (Pa)	tan δ	Thixotropy Area (Pa/s)	*G’G”* Crossover (Pa)	*G’* (Pa)	*G”* (Pa)	Tan δ
**Control (0:100)**	76.80 ± 8.50 ^a^	16.36 ± 0.09 ^d^	120.70 ± 8.60 ^d^	26.91 ± 3.39 ^d^	0.22 ± 0.00 ^c^	37.20 ± 1.20 ^d^	6.63 ± 0.23 ^c^	34.80 ± 8.70 ^c^	9.20 ± 2.00 ^c^	0.26 ± 0.00 ^c^
**A (10:90)**	436.30 ± 24.80 ^b^	15.69 ± 0.09 ^d^	119.70 ± 7.40 ^d^	38.97 ± 5.60 ^d^	0.33 ± 0.01 ^a^	181.70 ± 11.22 ^d^	7.78 ± 0.36 ^c^	138.40 ± 12.66 ^c^	50.70 ± 7.80 ^c^	0.37 ± 0.02 ^a^
**B (20:80)**	1510.70 ± 110.30 ^c^	61.09 ± 12.20 ^c^	541.40 ± 25.20 ^c^	159.40 ± 10.80 ^c^	0.29 ± 0.00 ^b^	373.50 ± 20.20 ^d^	27.18 ± 3.42 ^c^	402.50 ± 26.80 ^b,c^	126.40 ± 13.30 ^c^	0.31 ± 0.02 ^b^
**C (30:70)**	3722.00 ± 171.80 ^d^	226.47 ± 24.15 ^a^	1425.70 ± 50.00 ^a^	453.23 ± 22.20 ^a^	0.32 ± 0.01 ^a,b^	1398.30 ± 98.20 ^c^	44.41 ± 6.80 ^c^	954.10 ± 64.80 ^b^	326.80 ± 16.40 ^b^	0.34 ± 0.01 ^a,b^
**D (40:60)**	3347.70 ± 134.60 ^e^	183.97 ± 12.22 ^b^	1160.30 ± 39.40 ^b^	354.36 ± 18.80 ^b^	0.31 ± 0.01 ^a,b^	4021.10 ± 220.20 ^b^	179.97 ± 11.22 ^b^	3691.70 ± 412.00 ^a^	1267.00 ± 128.20 ^a^	0.34 ± 0.01 ^a,b^
**E (50:50)**	2481.70 ± 122.40 ^f^	174.62 ± 12.68 ^b^	1182.10 ± 38.80 ^b^	381.90 ± 19.40 ^b^	0.32 ± 0.01 ^a,b^	5022.00 ± 396.10 ^a^	456.20 ± 35.35 ^a^	3554.00 ± 368.80 ^a^	1172.30 ± 72.50 ^a^	0.33 ± 0.01 ^a,b^

POL-DAG: palm olein-based diacylglycerol oil; SBO: soybean oil. Data are expressed as the mean ± standard deviation. Mean values with different superscripts (a–f) in the same column are significantly different at *p* < 0.05 based on ANOVA and Tukey’s tests.
